# Exceptional Response to Multimodal Therapy in Stage IV Pancreatic Ductal Adenocarcinoma: A Case Report and Review

**DOI:** 10.7759/cureus.87044

**Published:** 2025-06-30

**Authors:** Carlos E Bonilla, Vaneza Ávila-Rodríguez, Mauricio F Acosta, Paola Jiménez-Vásquez, Silvia I Guerrero

**Affiliations:** 1 Gastrointestinal Cancer and Neuroendocrine Tumors Functional Unit, Cancer Treatment and Research Center (CTIC) Luis Carlos Sarmiento Angulo, Bogota, COL; 2 GIGA Research Group, Cancer Treatment and Research Center (CTIC) Luis Carlos Sarmiento Angulo, Bogota, COL; 3 Hospitalization Unit, Cancer Treatment and Research Center (CTIC) Luis Carlos Sarmiento Angulo, Bogotá, COL; 4 Diagnostic Imaging Functional Unit, Cancer Treatment and Research Center (CTIC) Luis Carlos Sarmiento Angulo, Bogota, COL

**Keywords:** chemotherapy for pdac and biliary tract carcinoma, metastasis directed therapy (mdt), pancreatic neoplasms, percutaneous microwave ablation, stereotactic radiosurgery srs

## Abstract

Pancreatic ductal adenocarcinoma (PDAC) is a highly aggressive malignancy with a dismal prognosis, frequently presenting with metastatic disease that drives its lethality. Conventional chemotherapy offers limited survival benefits, prompting exploration of novel treatment strategies, including metastasis-directed therapies (MDT) for oligometastatic PDAC, characterized by a limited number of metastatic lesions. We describe a 74-year-old woman with KRAS G12D-mutated PDAC and 5 liver metastases, exceeding typical oligometastatic criteria (≤3 lesions). Systemic therapy with modified fluorouracil, leucovorin, irinotecan, and oxaliplatin (modified FOLFIRINOX), followed by maintenance fluorouracil, leucovorin, and irinotecan (FOLFIRI), led to a partial response that induced an oligometastatic state. She subsequently underwent a multimodal non-surgical treatment sequence involving percutaneous microwave ablation (MWA) for hepatic metastases and stereotactic body radiotherapy (SBRT) to the primary pancreatic tumor, achieving complete radiologic resolution of all lesions. This yielded an exceptional ongoing 20-month progression-free survival, remarkable for a KRAS G12D-mutated PDAC, a subtype known for its challenging clinical course. Our literature review suggests that MDT, including percutaneous ablation techniques and SBRT, may improve outcomes in oligometastatic PDAC by enhancing local control and potentially by eliciting systemic immune-mediated effects. This case highlights the transformative potential of integrating tailored MDT with chemotherapy to deliver outstanding outcomes in select patients with advanced PDAC, though further research is needed to refine patient selection and optimize therapeutic approaches.

## Introduction

Pancreatic cancer is a major cause of cancer-related morbidity and mortality globally. Annually, it is estimated that approximately 510,992 new cases are diagnosed, resulting in 467,409 deaths. This ranks pancreatic cancer as the twelfth most common malignancy and the sixth leading cause of cancer death [1]. Its lethality is well-documented; data from the Surveillance, Epidemiology, and End Results (SEER) program in the United States show that the 5-year specific survival rate across all stages is only 12.8%, which decreases to 3.1% for patients diagnosed with metastatic disease [2]. This high mortality rate is due to its aggressive biological behavior, short latency period, and frequent diagnosis at advanced stages, often with metastases to the lymph nodes, liver, lungs, or peritoneum [3]. Liver metastases (LM) are strongly linked to a poor prognosis, as the tumor microenvironment in these lesions promotes an immunosuppressive state that, at least in part, affects clinical outcomes [4,5].

The management of advanced pancreatic cancer remains challenging due to the limited efficacy of therapeutic options and its resistance to conventional treatments. There is a subset of patients with limited metastatic disease, termed oligometastatic, for whom a multidisciplinary approach is essential. In some instances, these patients may benefit from therapies targeting both the metastatic lesions and the primary pancreatic tumor, potentially leading to improved clinical outcomes [6].

Presented here is the case of a patient with metastatic pancreatic adenocarcinoma harboring the pathogenic KRAS G12D mutation treated with combined chemotherapy, microwave ablation (MWA) of liver lesions, and stereotactic body radiotherapy (SBRT), achieving a complete clinical response. This case underscores the potential of multimodal therapies, contextualized here with a literature review of current advancements.

## Case presentation

A 74-year-old woman with hypertension, hypothyroidism, and type 2 diabetes presented with obstructive jaundice, epigastric and right upper quadrant pain radiating to the back, fatigue, loss of appetite, and unintentional weight loss. Contrast-enhanced magnetic resonance imaging (CE-MRI) and 2-deoxy-2-(¹⁸F)fluoro-D-glucose positron emission tomography-computed tomography ((¹⁸F)FDG PET-CT) revealed a 32 mm solid tumor in the pancreatic head and multifocal liver metastases, including at least 5 lesions up to 32 mm in diameter. An endoscopic ultrasound-guided biopsy of the pancreatic mass confirmed moderately differentiated pancreatic ductal adenocarcinoma (PDAC).

Genetic analysis by next-generation sequencing identified key mutations, including KRAS G12D (variant allele frequency (VAF) 12.6%), a SMAD4 splice site mutation (VAF 11%), and an ITPKB frameshift mutation (VAF 6.6%). The tumor exhibited a tumor mutational burden of 7.4 mutations per megabase, was negative for programmed death-ligand 1 (PD-L1) expression (<1%), and microsatellite instability (MSI) could not be assessed due to insufficient tumor content (<30%). The limited remaining tissue was also inadequate for additional immunohistochemistry (IHC) to evaluate mismatch repair (MMR) status. The disease was staged as IV (cT3N1M1).

In September 2023, she initiated first-line systemic treatment with fluorouracil, leucovorin, irinotecan, and oxaliplatin (modified FOLFIRINOX), completing 10 cycles by January 2024. This led to a partial response on imaging and a drop in cancer antigen 19-9 (CA 19-9) levels (from 12,000 U/mL to 2,088 U/mL). By February 2024, treatment was transitioned to maintenance fluorouracil, leucovorin, and irinotecan (FOLFIRI). Imaging in April and May 2024 demonstrated further tumor regression, with only three liver lesions remaining: one in segment V (14 mm, previously 32 mm) and two in segment VII (11 mm and 9 mm, previously 25 mm and 16 mm, respectively).

**Figure 1:**
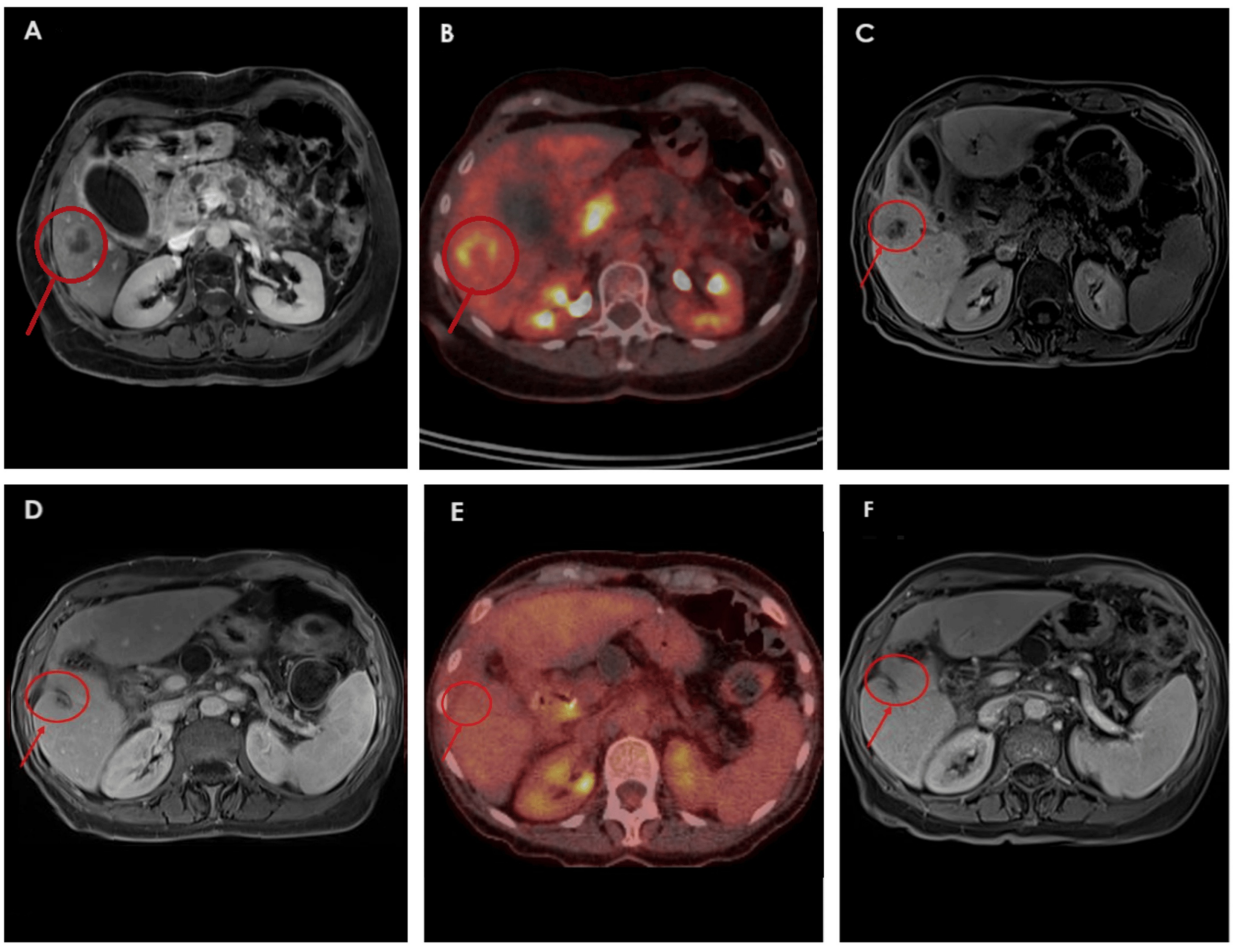
Sequential imaging of a segment V liver metastasis during systemic therapy and before and after microwave ablation (A) Axial T1-weighted CE-MRI from August 26, 2023, shows a well-defined 32 mm liver metastasis with peripheral contrast enhancement in segment V. (B) (¹⁸F)FDG PET-CT from August 29, 2023, demonstrates a hypermetabolic lesion in segment V of the liver, measuring approximately 32 mm with a necrotic center and a SUVmax of 7.1. (C) Axial T1-weighted CE-MRI from April 23, 2024, reveals a well-defined 14 mm hypointense lesion in segment V on a water-only Dixon VIBE sequence acquired 10 minutes after Primovist administration, consistent with a partially responding metastasis. (D) CE-MRI from October 21, 2024, shows a 10 mm lesion in segment V with low T1 signal intensity, no diffusion restriction, and no contrast enhancement—findings consistent with post-ablation changes and no evidence of residual tumor. (E) (¹⁸F)FDG PET-CT from October 15, 2024, shows no focal hypermetabolic activity in the area corresponding to the previously treated segment V lesion, consistent with a favorable post-ablation response. (F) CE-MRI from January 14, 2025, demonstrates a stable ablation zone with no evidence of recurrence or tumor viability, supporting a complete radiologic response to locoregional therapy. CE-MRI: contrast-enhanced magnetic resonance imaging; (¹⁸F)FDG PET-CT: 2-deoxy-2-(¹⁸F)fluoro-D-glucose positron emission tomography-computed tomography; SUVmax: maximum standardized uptake value

**Figure 2:**
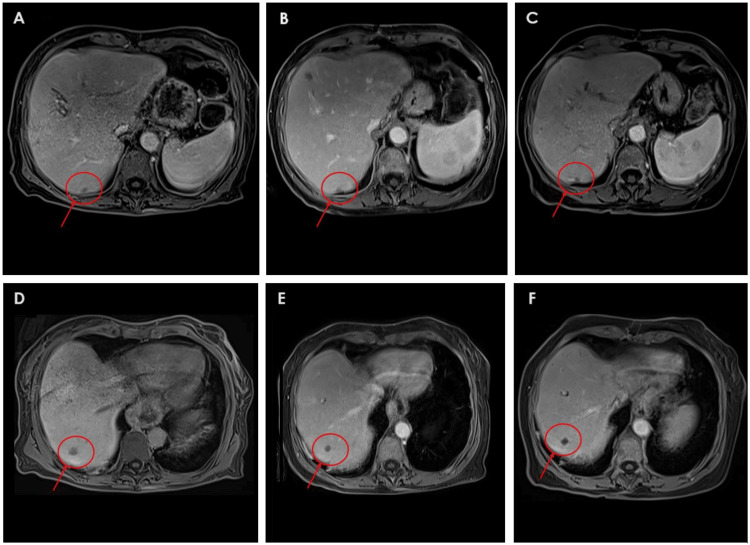
Sequential imaging of two hepatic metastases in segment VII during systemic treatment and before and after microwave ablation (A–C): Evolution of a subcapsular segment VII liver metastasis. (A) CE-MRI on April 23, 2024, shows an 11 mm hypointense hepatic metastatic lesion with low T1 signal intensity, intermediate T2 signal, and diffusion restriction, findings consistent with active disease. (B) CE-MRI on October 21, 2024, shows the lesion reduced to 6 mm, with intermediate T2 signal, low T1 signal, no diffusion restriction, and no contrast enhancement, again consistent with post-ablation changes and no signs of tumor viability. (C) CE-MRI on January 14, 2025, confirms a stable 6 mm treated lesion with no imaging evidence of recurrence. (D–F) Evolution of a second liver metastasis in segment VII. (D) CE-MRI on April 23, 2024, shows a 9 mm lesion (previously 13 mm) with low signal intensity on T1-weighted imaging and minimal contrast enhancement. (E) CE-MRI on October 21, 2024, reveals an 8 mm lesion with intermediate T2 signal, low T1 signal, no diffusion restriction, and no contrast enhancement, findings consistent with post-ablation changes and no evidence of tumor viability. (F) CE-MRI on January 14, 2025, shows a stable 8 mm lesion with identical signal characteristics, confirming the absence of tumor recurrence. CE-MRI: contrast-enhanced magnetic resonance imaging

Given her favorable response and the achievement of an induced oligometastatic state, a multidisciplinary tumor board recommended local ablative treatment. On June 5, 2024, she underwent successful MWA of the remaining liver lesions (60 W, 2 minutes per lesion), resulting in a further 90% reduction in CA 19-9 levels, from 2,088 to 114 U/mL. Maintenance therapy with FOLFIRI was continued. Follow-up CE-MRI showed no viable hepatic lesions and a 46% reduction in the size of the primary pancreatic tumor. Figures 1-2 illustrate the radiological evolution of the hepatic lesions in segments V and VII on CE-MRI and (¹⁸F)FDG PET-CT.

**Figure 3:**
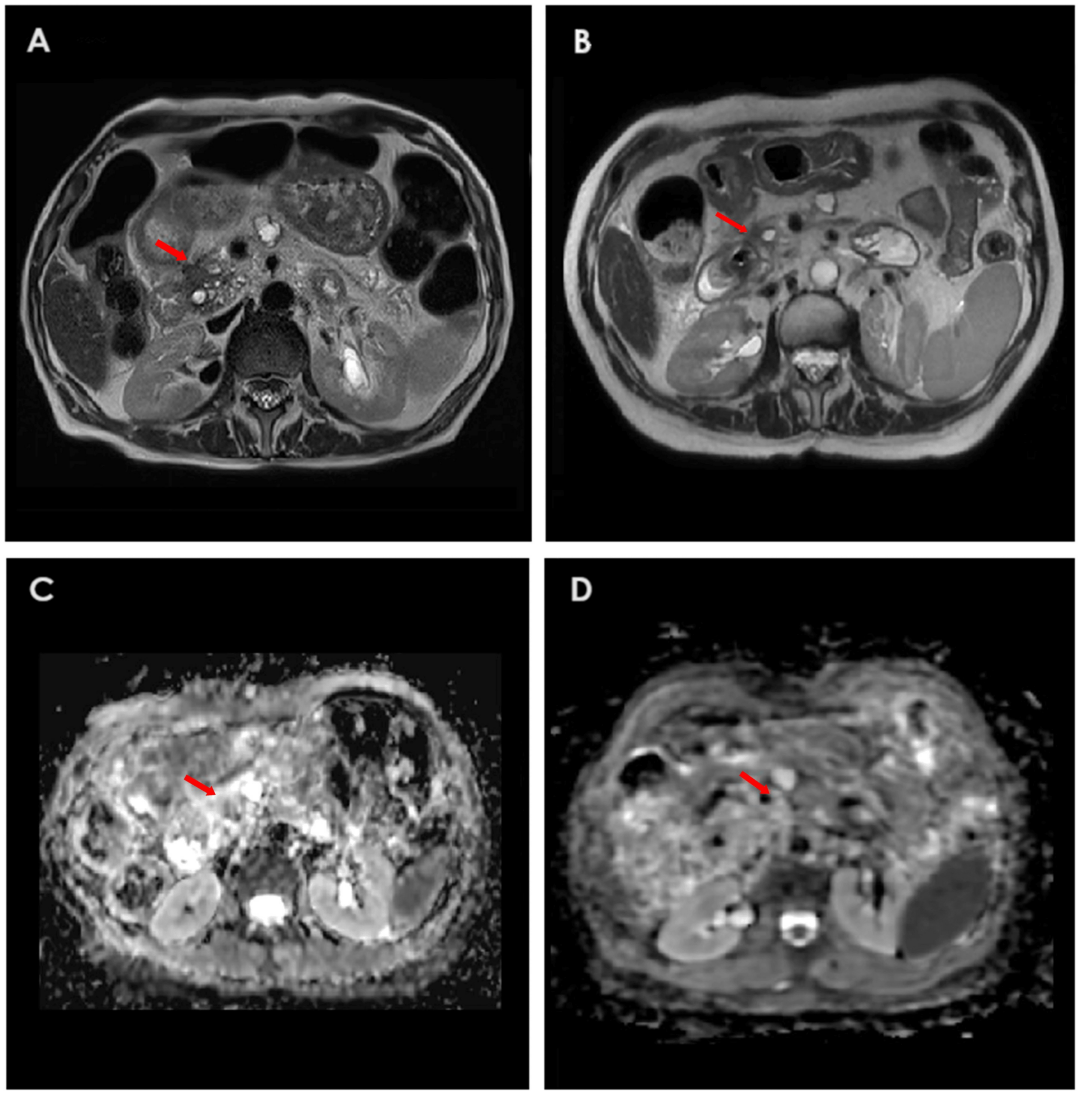
Abdominal CE-MRI images of the primary pancreatic tumor during systemic treatment and after pancreatic SBRT. (A) T2-weighted sequence on April 23, 2024, during chemotherapy, shows a 20 mm pancreatic head mass (red arrow) with ductal dilatation. (B) T2-weighted sequence on May 5, 2025, post-SBRT, demonstrates complete tumor resolution (red arrow) with moderate pancreatic atrophy. (C): ADC map on April 23, 2024, shows a pancreatic mass with diffusion restriction, indicating metabolic activity (viability). (D): ADC map on May 5, 2025, shows an absence of diffusion restriction in the lesion, consistent with non-viability of the tissue. CE-MRI: contrast-enhanced magnetic resonance imaging; SBRT: stereotactic body radiotherapy; ADC: apparent diffusion coefficient

**Figure 4:**
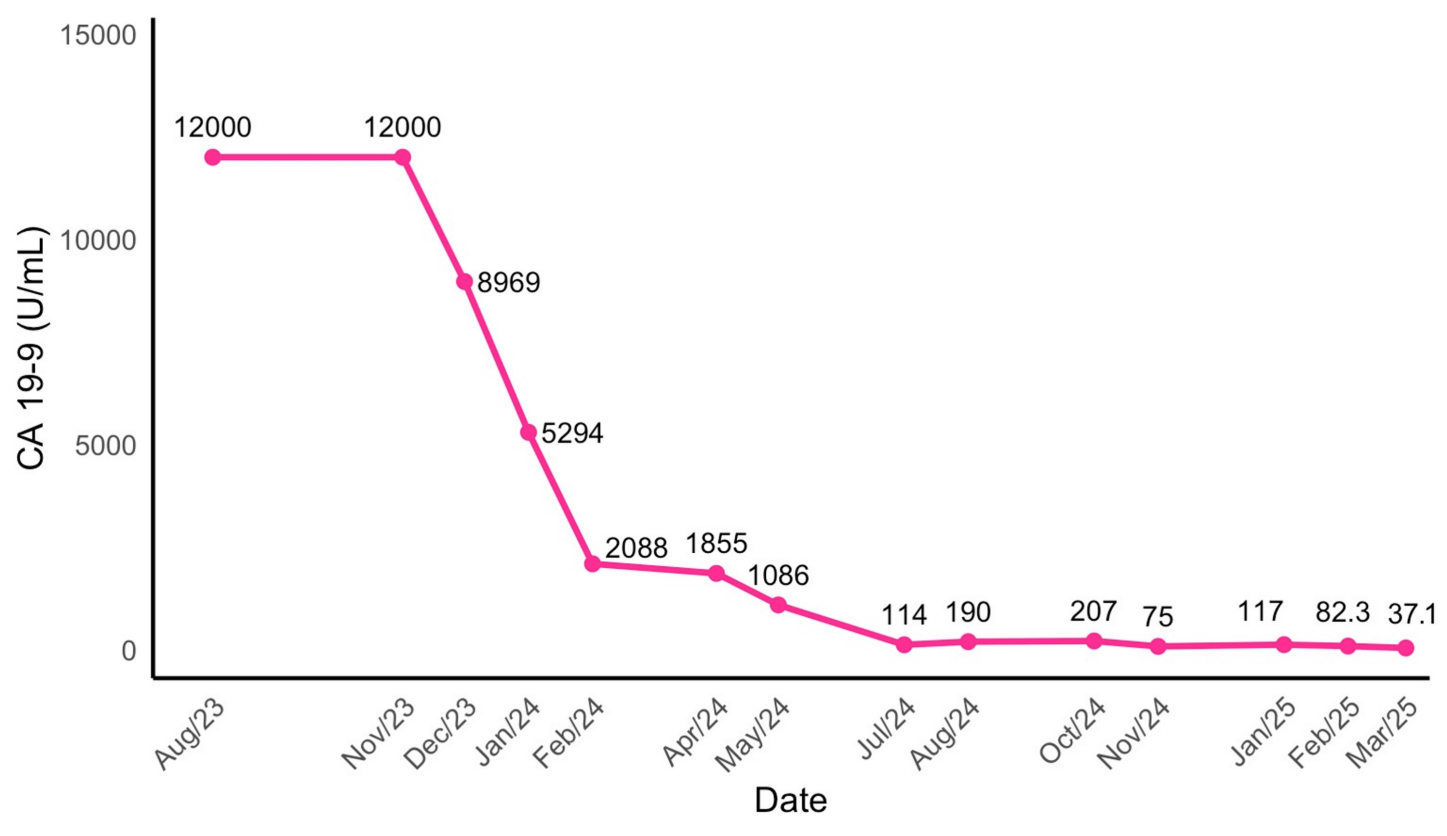
Temporal evolution of cancer antigen 19-9 (CA 19-9) during treatment The reference range for serum CA 19-9 levels is 0–39 U/mL.

Based on the absence of detectable metastatic disease on recent imaging and the continued decline in CA 19-9 levels, stereotactic body radiotherapy (SBRT) was offered to address the primary pancreatic tumor. She received a total of 25 Gy delivered in 5 fractions between January 20 and January 30, 2025. By March 2025, the CA 19-9 antigen had normalized. A follow-up abdominal CE-MRI in May 2025 confirmed a complete clinical response, with only post-ablation changes in the liver and resolution of the pancreatic mass. Figure 3 illustrates the resolution of the primary pancreatic tumor following SBRT, and Figure 4 shows the temporal evolution of CA 19-9 levels throughout the patient’s clinical course between August 2023 to March 2025, highlighting key changes in response to different therapeutic interventions.

The patient completed 22 cycles of maintenance FOLFIRI on April 30, 2025. Following documentation of a complete clinical response in all lesions, she was transitioned to maintenance capecitabine starting in May 2025. At the time of writing this report, she remains healthy, with full functionality, with an Eastern Cooperative Oncology Group (ECOG) performance status of 0, and at least 20 months of progression-free survival.

## Discussion

Pancreatic adenocarcinoma remains one of the most lethal malignancies, with limited improvements in survival despite advances in systemic therapy. Standard treatment for metastatic disease relies on systemic chemotherapy; the regimens most used and associated with the best outcomes are nanoliposomal irinotecan, 5-fluorouracil, and oxaliplatin (NALIRIFOX) or modified fluorouracil, irinotecan, and oxaliplatin (mFOLFIRINOX), with a median overall survival (OS) of 11.1 months in phase III trials [7,8]. Alternatively, gemcitabine plus nab-paclitaxel yields a median OS of 8.5 to 9.2 months [7,9]. Despite recent advances in systemic therapies, survival gains remain limited, emphasizing the need for novel and more effective therapeutic strategies such as metastasis-directed therapies (MDT) [10].

Within the spectrum of advanced pancreatic cancer, a subset of patients exhibits limited or oligometastatic disease, which may arise de novo or following initial systemic therapy. Although no universal consensus defines oligometastasis in this malignancy, a recent systematic review suggests it typically involves up to three metastatic lesions in a single extrapancreatic organ; however, some studies extend this definition to include up to five metastases [5]. Identifying these patients is key, as they might benefit not only from conventional systemic therapies but also from targeted treatments addressing both metastases and the primary tumor, potentially improving clinical outcomes. The most common metastatic sites in pancreatic cancer include the liver (75-80%), peritoneum (13-30%), lungs (15-18%), and extraregional lymph nodes (12%) [3]. Liver metastases (LMs) stand out for their particularly poor prognosis. An analysis of the SEER program in the US, encompassing 13,233 patients, demonstrated that LMs are associated with reduced OS compared to other metastatic sites, such as lung or distant lymph nodes [4].

In pancreatic adenocarcinoma and other cancers, LMs create a locally and systemically immunosuppressive microenvironment that worsens patient outcomes [9-11]. The liver hosts a complex immune network involving hepatic stellate cells (HSCs), liver sinusoidal endothelial cells (LSECs), Kupffer cells (KCs), regulatory T (Treg) cells, dendritic cells (DCs), and myeloid-derived suppressor cells (MDSCs), all contributing to immune tolerance [11,12]. Studies show that LM weaken CD4+ and CD8+ T-cell function, forming an “immune desert” through mechanisms like hepatic macrophage-induced apoptosis via Fas-FasL [11] and programmed death-ligand 1 (PD-L1) expression by LSECs, KCs, and HSCs, which interacts with programmed death-1 (PD-1) [12]. HSCs also secrete transforming growth factor-β (TGFβ), promoting MDSCs-cells that suppress immune responses [12]. Similarly, KCs, the liver’s resident macrophages, express PD-L1 and reduce major histocompatibility complex (MHC) expression on LSECs, limiting immune activation [12]. Hepatocytes add to this suppression, as interleukin-6 (IL-6)/signal transducer and activator of transcription 3 (STAT3) signaling leads them to release serum amyloid A (SAA) proteins, which, in vitro, block DC differentiation, reducing CD8+ T-cell infiltration and immune surveillance [13]. Treg cells further dampen immunity by expressing CD25 and cytotoxic T lymphocyte protein 4 (CTLA4), releasing cytokines like TGFβ and IL-10, and consuming lipids and glucose, all of which impair tumor-killing T cells [12].

Hepatic DCs, especially the plasmacytoid subtype, respond weakly to toll-like receptor (TLR) stimulation and lower costimulatory molecules, promoting tolerance [12]. Meanwhile, hepatic myofibroblasts (HMF), expressing alpha-smooth muscle actin, secrete mediators and matrix components that support tumor growth and form a dense stroma, blocking immune cells and drug delivery while inducing suppression via PD-1/PD-L1 and other pathways [14]. Disrupting this tolerogenic environment with metastasis-directed therapies (MDT) may boost antitumor immune responses.

**Figure 5:**
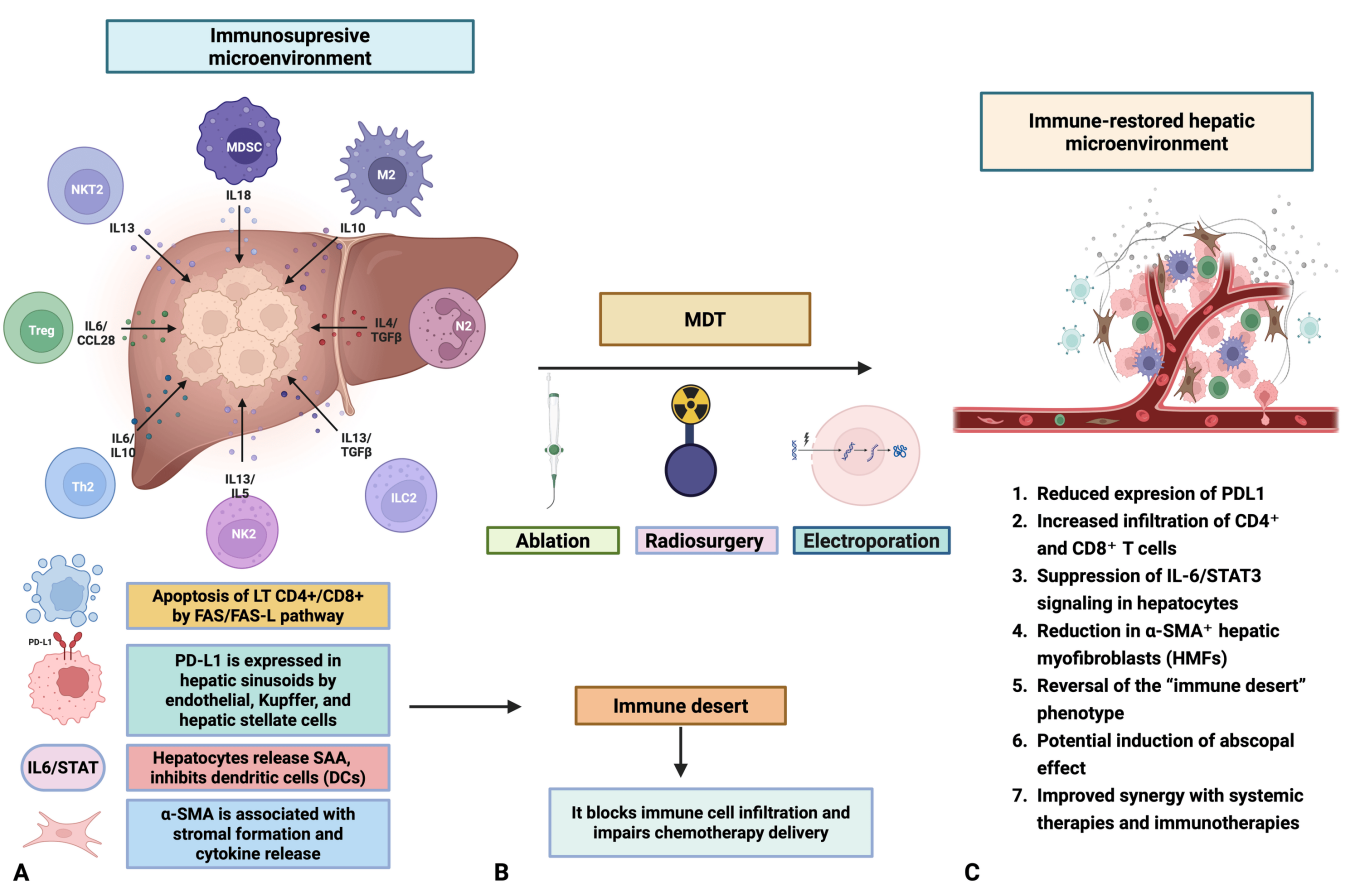
Hepatic metastatic microenvironment in pancreatic ductal adenocarcinoma (PDAC) and potential immunologic modulation following metastasis-directed therapy (MDT) This figure illustrates the immune landscape of liver metastases from PDAC and how it may be reshaped by MDTs, including ablation, stereotactic radiosurgery, and irreversible electroporation (IRE). A) Immunosuppressive microenvironment: Before treatment, the hepatic metastatic niche is dominated by a tolerogenic immune profile that promotes tumor progression. Immunoregulatory cells—Myeloid-Derived Suppressor Cells (MDSCs), M2 macrophages, Th2 cells, Tregs, NKT2, ILC2, and NK2—release cytokines (IL-6, IL-10, IL-13, IL-18, TGF-β) that drive immunosuppression, tissue remodeling, and angiogenesis. The liver acts as an immune sink, attracting circulating cytotoxic T cells and inducing their apoptosis, which contributes to systemic T-cell depletion. Sinusoidal cells (endothelial, Kupffer, stellate) express PD-L1, promoting T-cell exhaustion and FasL-Fas-mediated apoptosis. IL-6/STAT3-activated hepatocytes secrete serum amyloid A (SAA), impairing dendritic cell function. α-SMA⁺ myofibroblasts reinforce stromal immunosuppression through a fibrotic, cytokinerich matrix. The result is an “immune desert” that impedes T-cell infiltration and limits therapeutic efficacy. B) Therapeutic intervention: MDTs exert local cytotoxicity and may induce systemic immune effects by disrupting stroma, altering cytokines, and releasing tumor antigens. This may reprogram the microenvironment toward an immune-permissive state. C) Immune-restored microenvironment: Post-treatment, PD-L1 expression, α-SMA⁺ fibroblasts, and IL-6/STAT3 signaling are reduced. This allows greater CD4⁺ and CD8⁺ T-cell infiltration and reversal of the immune desert. Such remodeling may enhance responses to systemic therapy and checkpoint inhibitors, and potentially trigger the abscopal effect. Image created using BioRender (https://www.biorender.com).

In oligometastatic pancreatic cancer, MDT, such as thermal ablation, stereotactic radiosurgery (SRS), and irreversible electroporation (IRE), could potentially not only eradicate macroscopically visible metastatic disease but also provide an important additional benefit. These approaches might disrupt the tolerogenic microenvironment, restore antitumor immunity, and possibly trigger an abscopal effect, potentially contributing to improved disease control in patients with limited metastatic burden [15]. Figure 5 illustrates the immunosuppressive hepatic metastatic microenvironment in PDAC and its transformation following MDT, highlighting the potential for immune restoration and enhanced therapeutic outcomes.

Emerging evidence supports MDT’s potential in oligometastatic PDAC. Elamir et al. reported a doubled median OS (≈2 years versus 1 year) with stereotactic ablative radiotherapy (SABR) in 20 patients with one to five metastatic lesions, alongside extended chemotherapy breaks (>6 months in many cases) [16]. Ikuta et al. described a case where a liver-first approach using percutaneous MWA, followed by chemotherapy and primary tumor resection, resulted in over a year of disease-free survival, suggesting effective local control [17]. Liu et al. demonstrated that combining irreversible electroporation (IRE) with systemic chemotherapy in unresectable pancreatic ductal adenocarcinoma (PDAC) significantly improved median progression-free survival (PFS) and overall survival (OS) compared to IRE alone, with manageable complications [18]. These findings highlight the value of integrating locoregional procedures like IRE with systemic therapy to enhance outcomes and support its potential role in downstaging.

A retrospective propensity score-matched study by Yan et al. compared chemotherapy alone versus a combination of chemotherapy and thermal ablation in patients with PDAC and liver metastases. The study found that patients who received the combined treatment had significantly longer overall survival than those treated with chemotherapy alone (median OS 10.77 vs. 5.77 months before matching; 8.17 vs. 5.77 months after matching). Multivariate analysis confirmed that the addition of ablation was an independent prognostic factor. These findings suggest that integrating ablation with chemotherapy may enhance outcomes in patients with metastatic disease [19]. Erdem et al. reviewed ablative techniques - radiation, radiofrequency ablation (RFA), MWA, and IRE - noting their potential to stimulate antitumor immunity (e.g., via the abscopal effects) [15].

The strongest evidence to date supporting MDT comes from the EXTEND trial, a multicenter, randomized phase II study that evaluated MDT - primarily SRS (e.g., 50 Gy in 4 fractions or 70 Gy in 10 fractions) and some thermal ablation - combined with systemic therapy versus systemic therapy alone in 40 patients with up to 5 metastatic lesions. MDT significantly prolonged median PFS from 2.5 to 10.3 months, with a hazard ratio (HR) of 0.43 (95% CI, 0.20 to 0.94) and P = 0.030 for the stratified log-rank test, with no severe adverse events reported. Translational analyses demonstrated increased activation and the proliferation of CD8+ T-cells, likely driven by tumor cell damage and neoantigen release, suggesting systemic immune priming that may augment cytotoxic T-cell responses and improve overall survival [20]. These findings, along with previous studies, suggest that MDT, including SBRT, thermal ablation, and IRE, can delay progression, extend survival, and reduce dependence on chemotherapy in selected patients with oligometastatic PDAC. Table 1 summarizes the progression-free survival (PFS) and overall survival (OS) outcomes from major phase 3 trials investigating systemic therapies, alongside studies exploring multimodal approaches incorporating locoregional treatment of oligometastases, highlighting their impact on clinical outcomes and relevance to the current case.

**Table 1:** Comparative analysis of progression-free survival (PFS) and overall survival (OS) in Phase 3 systemic therapy trials and multimodal oligometastatic treatment studies for pancreatic cancer * Studies with patients with oligometastatic disease. NALIRIFOX: nanoliposomal irinotecan, 5-fluorouracil, and oxaliplatin; FOLFIRINOX: fluorouracil, leucovorin, irinotecan, and oxaliplatin; FOLFIRI: fluorouracil, leucovorin, and irinotecan; SBRT: stereotactic body radiotherapy; MDT: metastasis-directed therapy; MWA: microwave ablation; IRE: irreversible electroporation

Study (Reference)	Study Type	Therapeutic Approach	Median PFS (months)	Median OS (months)	OS Hazard Ratio (95% CI); P
NAPOLI-3 (Wainberg ZA, et al., 2023) [7]	Phase 3 RCT	NALIRIFOX vs. Gem+Nab-Paclitaxel	7.4 vs. 5.6	11.1 vs. 9.2	HR 0.83 (95% CI, 0.70 to 0.99); P=0.036
PRODIGE-4 / ACCORD-11 (Conroy T, et al., 2011) [8]	Phase 3 RCT	FOLFIRINOX vs. Gemcitabine	6.4 vs. 3.3	11.1 vs. 6.8	HR 0.57 (95% CI, 0.45 to 0.73); P< 0.001
MPACT (Von Hoff DD, et al., 2013) [9]	Phase 3 RCT	Nab-Paclitaxel + Gem vs. Gem	5.5 vs. 3.7	8.5 vs. 6.7	HR 0.72 (95% CI, 0.62 to 0.83); P<0.001
EXTEND Trial * (Ludmir EB et al., 2024) [20]	Phase 2 RCT	MDT + Chemotherapy vs. Chemotherapy	10.3 vs. 2.5	12 vs. 10	HR 0.58 (95% CI, 0.25 to 1.34); P=0.2
Liu B, et al., 2019 * [18]	Prospective	IRE + Chemo vs. IRE	11.7 vs. 9.45	13.56 vs. 11.6	P=0.0398
Elamir AM et al., 2022* [16]	Retrospective Cohort	SBRT + Chemotherapy vs. Chemotherapy	40 vs. 14	42 vs. 18	HR 0.21 (95% CI, 0.08 to 0.53); P=0.0003
Yan X, et al., 2021* [19]	Retrospective	Chemo + Thermal Ablation vs. Chemo	NR	10.77 vs. 5.77	P=0.011
Ikuta S, et al., 2023* [17]	Case Report	Thermal ablation → FOLFIRINOX → Surgery → FOLFIRINOX	20+	20+	N/A
Current case, Bonilla C, et al., 2025*	Case Report	mFOLFIRINOX → FOLFIRI + MWA to liver metastases → pancreatic SBRT → FOLFIRI	20+	20+	N/A

This case report has limitations, including a potential selection bias due to the patient’s unique presentation. The exceptionally favorable outcome may not apply broadly to all KRAS G12D-mutated PDAC patients due to individual variability. These findings underscore the need for larger studies to validate the efficacy of metastasis-directed therapies in patients with advanced PDAC.

## Conclusions

This case of a 74-year-old woman with KRAS G12D-mutated pancreatic ductal adenocarcinoma (PDAC) and 5 liver metastases at presentation is distinguished by its exceptional clinical course and therapeutic approach. Initially exceeding the typical criteria for oligometastatic disease (≤3 lesions), her disease transitioned to an induced oligometastatic state following systemic therapy with modified fluorouracil, irinotecan, and oxaliplatin (mFOLFIRINOX) and maintenance FOLFIRI. This response enabled the use of microwave ablation (MWA) for her hepatic metastases and stereotactic body radiotherapy (SBRT) to the primary tumor. This multimodal strategy resulted in a complete clinical response and an outstanding ongoing 20-month progression-free survival, a particularly remarkable outcome given the typically aggressive course observed in patients with KRAS mutations. Her favorable result aligns with findings and other emerging evidence, reinforcing the value of carefully integrating local therapies to enhance disease control and prolong survival in select patients with oligometastatic PDAC. While this case provides valuable insights, it is important to acknowledge that, as a single case report, it is subject to selection bias and limited generalizability. Continued research is essential to better define and optimize the role of multidisciplinary strategies, such as SBRT, RFA, and IRE, in addressing the formidable challenges of this disease.
